# Risk factors and pattern of weight gain in youths using antipsychotic drugs

**DOI:** 10.1007/s00787-020-01614-4

**Published:** 2020-08-24

**Authors:** Casper C. L. van der Esch, Sanne M. Kloosterboer, Jan van der Ende, Catrien G. Reichart, Mirjam E. J. Kouijzer, Matthias M. J. de Kroon, Emma van Daalen, Wietske A. Ester, Rob Rieken, Gwen C. Dieleman, Manon H. J. Hillegers, Teun van Gelder, Birgit C. P. Koch, Bram Dierckx

**Affiliations:** 1 Department of Hospital Pharmacy, Erasmus MC, University Medical Center, Rotterdam, The Netherlands; 2 Department of Child and Adolescent Psychiatry/Psychology, Erasmus MC, University Medical Center, Rotterdam, The Netherlands; 3 Department of Child and Adolescent Psychiatry, Curium-LUMC, Leiden University Medical Center, Oegstgeest, The Netherlands; 4 GGz Breburg, Centre of Youth, Breda, The Netherlands; 5 de Kroon Child Psychiatry, Breda, The Netherlands; 6 Yulius Mental Health, Dordrecht, The Netherlands; 7 Sarr Expert Centre for Autism, Youz Child and Adolescent Psychiatry, Rotterdam, The Netherlands; 8 Parnassia Psychiatric Institute, The Hague, The Netherlands; 9 Department of Youth, GGZ Delfland, Delft, The Netherlands

**Keywords:** Antipsychotics, Weight gain, Body mass index, Risk factors, Child, Adolescent

## Abstract

Antipsychotic-induced weight gain is a major health concern in children and adolescents. The aim of this study was to identify risk factors for weight gain during short-, middle- and long-term treatment with antipsychotic drugs in this young population. We analysed a combined prospective and a retrospective observational cohort of Dutch children and adolescents, starting with risperidone, aripiprazole or pipamperone treatment. Linear mixed models were used to test whether sex, age, baseline body-mass-index (BMI) *z *score, type of antipsychotic, dose equivalent/kg, duration of use, previous antipsychotic use, ethnicity, physical exercise, IQ, concomitant medication, and psychiatric classification predicted the BMI *z *score for a follow-up of < 15 weeks, 15–52 weeks or > 52 weeks. A total of 144 patients were included with a median [interquartile range ([IQR)] age of 9 (4) years and median follow-up of 30 (73) weeks. During the complete follow-up, the median (IQR) weight gain was 0.37 (0.95) BMI *z *score points. Antipsychotic-induced weight gain was found to be most pronounced during the first 15 weeks of use (BMI *z *score increase per week *β* = 0.02, 95% CI 0.01–0.03, *p* = 0.002). A higher baseline BMI *z *score and the absence of stimulant use were associated with a higher BMI *z *score during the entire follow-up and after 15 weeks, respectively. Previous treatment with an antipsychotic drug was associated with less weight gain during the first 15 weeks of treatment. Our findings underscore the importance of close patient monitoring during the first weeks of antipsychotic treatment with a focus on patients with a high baseline BMI *z *score.

## Introduction

Antipsychotic-induced weight gain has been recognized as a major health concern in children and adolescents [1]. Although the magnitude of weight gain differs across type of antipsychotics and individuals, on average, 1 in 7 minors gains 7% or more weight within the first 6–8 weeks of treatment [2]. This is not only highly stigmatizing, but also involves serious long-term health risks. Weight gain is associated with glucose and lipid abnormalities, thereby increasing the risk for diabetes and cardiovascular morbidity in children and adolescents using antipsychotics [3, 4]. It has been suggested that this leads to higher rates of unexpected death in this population, even at young age [5].

The observed weight gain in youths starting antipsychotic treatment is highly heterogeneous, with some youths gaining a lot of weight, while others do not [6]. This heterogeneity suggests that certain patient-related factors underlie the risk for antipsychotic-induced weight gain. The identification of these risk factors is an important target in the prevention of obesity, as it can facilitate early recognition and interventions for children and adolescents at risk. Such interventions can include both behavioural and pharmacological interventions, which are proven to be effective at least to some extent in the management of antipsychotic-related weight gain in this population [6, 7].

To date, literature is inconclusive about which children and adolescents are particularly at risk for weight gain during antipsychotic treatment. While it has been suggested that girls are at higher risk than boys [4], other studies have found the opposite [8], or did not find an influence of gender at all [9]. Likewise, some studies showed that a young age is associated with more weight gain [10, 11], while others found older age increases the risk for obesity [4]. Data on clinical predictors are generally limited, although several studies found that a low baseline body mass index (BMI) is associated with more weight gain [8, 12], and the concomitant use of stimulants would not be of significant influence [12, 13].

It can be hypothesized that, amongst other factors, different follow-up durations might have contributed to these mixed findings. Weight gain in children and adolescents has been reported to be most pronounced during the first weeks of antipsychotic use and to stabilize during continued treatment, although long-term data are limited [14, 15]. In these different phases of weight acceleration, several mechanisms that are involved in antipsychotic-related weight gain are likely to contribute differently. Although these mechanisms are only poorly understood, several neurotransmitter and endocrine systems, such as serotonergic, dopaminergic and histaminergic receptors and leptin, have been implicated [16, 17]. As different patient-related factors, such as sex or comedication, might influence these mechanisms individually, the influence of risk factors might be time-dependent as well.

Therefore, the aim of this study is to describe risk factors for weight gain in children and adolescents using antipsychotic drugs with a short-, middle- and long-term duration of use.

## Methods

### Population

The study population consisted of children and adolescents treated with risperidone, aripiprazole, or pipamperone in the southwest region of the Netherlands. These three antipsychotics are the most frequently prescribed antipsychotics in this population in the Netherlands [18].

The study population consisted of a prospective and a retrospective observational cohort. The prospective cohort included children and adolescents that were enrolled in an observational Dutch multicentre trial (NTR 6050) with a follow-up of 6 months. These patients were treated in an inpatient or outpatient setting in the Erasmus Medical Center, or one of 6 other participating centres in the southwest region of the Netherlands (1 other academic tertiary-care centre and 5 psychiatric secondary-care centres) between August 2016 and November 2018. All patients and/or their legal representatives gave written informed consent before entering the study. The study was approved by the medical ethical committee of the Erasmus Medical Centre, the Netherlands (number MEC-2016-124). The retrospective cohort consisted of children and adolescents being treated in the Erasmus medical centre between 1 January 2012 and 31 December 2017. The medical ethical committee of the Erasmus Medical Centre waived informed consent for this study (MEC-2018-1613).

Patients were included based on the following criteria: [1] Treatment with risperidone, aripiprazole or pipamperone, [2] No simultaneous use of other antipsychotics, [3] Bodyweight known at least 2 weeks before or after the start of antipsychotic, [4] Minimally 1 other bodyweight known during use of antipsychotic, [5] Age up to 18 years, [6] No concomitant condition with direct influence on bodyweight (e.g. eating disorder, Prader–Willi syndrome). Patients who had used more than one antipsychotic during the study periods, were only included with the first antipsychotic that met the inclusion criteria.

### Outcome

The outcome was age- and gender-specific BMI *z *score. To calculate the BMI *z *score, BMI values were transformed into BMI *z *scores based on the World Health Organisation (WHO) BMI for age reference values (5–19 years) [19]. According to the WHO, a BMI *z* score > 1 is considered overweight, and a BMI *z *score > 2 is considered obesity. In the prospective cohort, weight and height were measured at baseline and at 6 months, and for a subset of patients that initiated antipsychotic treatment at start of the study, also at 1 month and at 3 months. In the retrospective cohort, weight and height were measured at variable time points during visits as part of routine clinical care. When a height measurement was missing but weight was known, a height measurement of the same patient that was performed within 5 weeks from that time point could be used. An average child or adolescent grows 6 cm per year, so 5 weeks would amount to maximal ± 0.6 cm difference in height which was considered insignificant [20].

### Predictors

Date of birth, sex, IQ and psychiatric classification according to the Diagnostic and Statistical Manual of Mental Disorders (DSM) IV or V were retrieved from the medical files. Physical exercise, country of birth of mother, father and child were collected based on questionnaires that were part of routine clinical care. For the antipsychotic drugs, the following data were collected: type, dose, start date, stop date and previous use of antipsychotics. Dose equivalent/kg was calculated based on defined daily doses from the WHO which were multiplied by 100 [21]. The dose equivalents were 5 mg for risperidone, 15 mg for aripiprazole and 200 mg for pipamperone. IQ was treated as a binary variable, defined as intellectual disability (IQ < 70) or not (IQ > 70). If IQ-scores were not available, medical records were screened for the diagnosis of intellectual disability. Ethnicity was considered a binary variable, and scored as either both parents were born in the Netherlands, or not. Concomitant stimulant use was defined as the use of methylphenidate or amphetamines at time of bodyweight and height measurement.

### Statistical analysis

First, the course of weight gain within the sample was visually inspected. As the slope of the BMI *z *score gain differed during the first 15 weeks of treatment (short term), after 15 weeks (medium term) and after 52 weeks (long term), three separate analyses were performed for these time frames as described below. These time frames were considered clinically relevant, as randomized controlled trials have shown that weight gain is significant during the first weeks of use [2] and many children use antipsychotic drugs only for a short period of time, but at the same time, a considerable proportion of children use antipsychotic drugs for more than a year [18].

The data were analysed per time frame using linear mixed models with visits clustered within patients. The BMI *z *score values at different time points during follow-up were used as dependent variable. Multiple imputation was used to replace missing values for IQ (4% missing), physical exercise (15% missing) and ethnicity (10% missing). Imputation was based on the baseline data, as these variables did not change over time, and data were imputed on the individual level only. The fully conditional specification procedure in SPSS was used to impute five datasets for the analyses [22]. The imputed datasets were augmented with the repeated measurements to conduct the linear mixed models. The validity of the imputed variables was checked by computing the observed and imputed frequencies of the scores; there were no large differences between these frequencies. Subsequently, the assumptions of the linear mixed model were checked with visual inspection for the three time frames separately, and were all met [23]. First, all predictors were analysed in a univariate model as fixed effect with random intercept together with baseline BMI *z *score (model 1). For the predictors with *p* < 0.15 in the univariate model, interaction terms with time were added (model 2) to test whether they also predicted the BMI *z *score increase per week (rather than for the whole time frame). The predictors and interaction terms that were statistically significant (*p* < 0.05) in one or more time frames were combined in a multivariate model per time frame (model 3). Variables with a *p *value < 0.05 in one or more time frames were selected for the final model. SPSS Version 25 (SPSS Inc., Chicago, IL, USA) was used for the analyses.

## Results

### Sample

A total of 289 unique patients were screened for inclusion in the retrospective cohort, of which 90 patients were included. The main reasons for exclusion were: no use of risperidone, aripiprazole or pipamperone (*n* = 57), no bodyweight known at baseline (*n* = 66), simultaneous use of other antipsychotics (*n* = 15), and no bodyweight measurements at follow-up (*n* = 15). An additional number of 54 patients were included in the prospective cohort, resulting in a total study sample of 144 unique patients. Within this sample, 18.8% of patients were overweight and 11.8% of patients were obese at start of antipsychotic treatment. The patient characteristics can be found in Table 1.

**Table 1:** Patient characteristics

Baseline BMI *z *score	0.23 (1.91)
Age, years	9 (4)
Male, *n* (%)	109 (76)
Dutch nationality^a^, *n* (%)	90 (70)
Follow-up, weeks	30 (72.5)
No. of visits	4 (4)
Antipsychotic, *n* (%)	
Risperidone	93 (65)
Aripiprazole	22 (15)
Pipamperone	29 (20)
Previous antipsychotic use, *n* (%)	60 (42)
Psychiatric diagnosis, *n* (%)	
ASS	124 (86)
ADHD	68 (47)
Other	43 (30)
Comorbidities, *n* (%)	
Epilepsy	11 (8)
Other	78 (54)
IQ < 70^a^, *n* (%)	45 (33)
Physical exercise^a^, *n* (%)	57 (47)

Total *n* = 144 children and adolescents. All variables are presented as medians (interquartile range, IQR) unless otherwise stated

^a^Available data: nationality *n* = 129 (90% complete), IQ *n* = 138 (96% complete), physical exercise *n* = 122 (85% complete)

### BMI z score

**Fig. 1:**
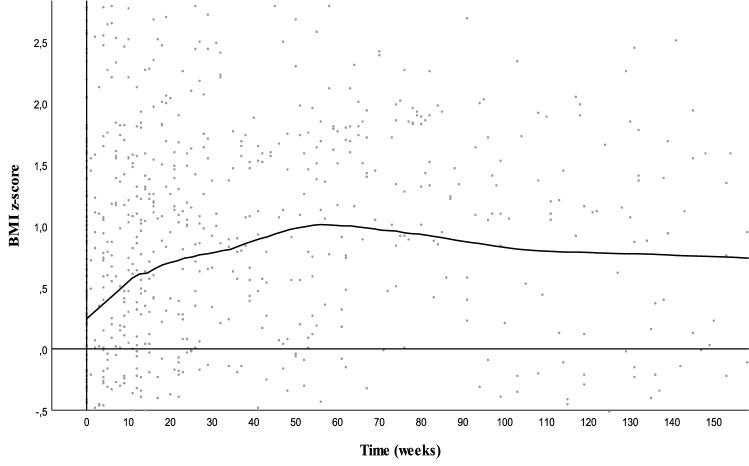
Pattern of weight gain

During the complete follow-up, the median (interquartile range, IQR) weight gain was 0.37 (0.95) BMI *z *score points. The increase in BMI *z *score was most pronounced during the first 15 weeks of treatment, followed by a slower increase up to 52 weeks and a slight decrease after 52 weeks, which is shown in Fig. 1. Analyses were separately done for the time frames < 15 weeks (short term, *n* = 144 patients), 15–52 weeks (middle term, *n* = 111 patients) and > 52 weeks (long term, *n* = 58 patients) duration of use.

When predictors were individually tested together with only baseline BMI *z *score as a covariate, dose equivalent/kg, duration of use, sex, no previous antipsychotic use, and stimulant use were significantly correlated with BMI *z *score during one or more follow-up time frames (see Table 2, model 1). With addition of interaction terms with time to these models, only a higher dose equivalent/kg (*β* = 0.02, 95% CI 0.01–0.04, *p* = 0.011), a lower baseline BMI *z *score (*β* = − 0.01, 95% CI − 0.02 to − 0.01, *p* < 0.001) and no previous antipsychotic treatment (*β* = 0.02, 95% CI > 0.00 to 0.03, *p* = 0.01) were found to significantly predict the BMI *z *score increase per week, during the first 15 weeks of use (Table 2, model 2). The absence of stimulant use was significantly associated with a higher mean BMI z-score during the first 15 weeks, as was a higher baseline BMI z-score during all time frames.

**Table 2:** Predictors of BMI *z*-score in children and adolescents during antipsychotic treatment

	< 15 weeks follow-up^a^				15–52 weeks follow-up^a^				> 52 weeks follow-up^a^			
	*β*	*p* value	SE	95% CI	*β*	*p *value	SE	95% CI	*β*	*p* value	SE	95% CI
Model 1												
Dose equivalent/kg	**0.27**	** < 0.001**	**0.06**	**0.16 to 0.38**	0.55	0.316	0.05	− 0.05 to 0.16	− 0.05	0.307	0.05	− 0.14 to 0.04
Duration of use (weeks)	**0.03**	** < 0.001**	**0.00**	**0.03 to 0.04**	**0.01**	**0.088**	**0.00**	**0.00 to 0.02**	**0.00**	** < 0.001**	**0.00**	**0.00 to 0.00**
Baseline BMI *z *score	**0.92**	** < 0.001**	**0.02**	**0.90 to 0.96**	**0.80**	** < 0.001**	**0.05**	**0.7 to 0.9**	**0.69**	** < 0.001**	**0.07**	**0.56 to 0.83**
Age (years)	0.00	0.661	0.00	0.00 to 0.00	0.00	0.507	0.00	0.00 to 0.00	0.00	0.457	0.00	0.00 to 0.00
Sex (male)	**0.10**	**0.076**	**0.06**	− **0.01 to 0.22**	**0.27**	**0.096**	**0.16**	− **0.05to 0.60**	0.22	0.328	0.22	− 0.22 to 0.66
Antipsychotic												
Risperidone	0.07	0.197	0.05	− 0.04 to 0.18	0.16	0.309	0.15	− 0.14 to 0.45	− 0.07	0.756	0.22	− 0.50 to 0.36
Aripiprazole	− 0.03	0.401	0.07	− 0.17 to 0.12	0.05	0.814	0.20	− 0.35 to 0.45	0.16	0.950	0.26	− 0.49 to 0.52
Pipamperone	− 0.07	0.255	0.06	− 0.19 to 0.05	− 0.26	0.155	0.18	− 0.61 to 0.10	0.08	0.762	0.26	− 0.43 to 0.59
No previous antipsych. treatment	**0.11**	**0.026**	**0.05**	**0.01 to 0.21**	0.16	0.273	0.14	− 0.12 to 0.43	0.13	0.524	0.20	− 0.27 to 0.53
Psychiatric classification												
ASS	− 0.01	0.913	0.07	− 0.15 to 0.14	0.06	0.804	0.23	− 0.39 to 0.50	0.27	0.328	0.27	− 0.27 to 0.80
ADHD	− 0.01	0.786	0.05	− 0.12 to 0.09	0.02	0.905	0.15	− 0.27 to 0.31	0.23	0.249	0.20	− 0.16 to 0.62
Other psychiatric class	0.03	0.596	0.05	− 0.08 to 0.14	− 0.15	0.339	0.15	− 0.45 to 0.15	− 0.10	0.630	0.20	− 0.49 to 0.29
Somatic diagnosis												
Epilepsy	− 0.10	0.318	0.10	− 0.29 to 0.09	− 0.16	0.534	0.26	− 0.66 to 0.34	− 0.48	0.115	0.31	− 1.08 to 0.12
Other	− 0.05	0.325	0.05	− 0.15 to 0.05	− 0.01	0.924	0.14	− 0.28 to 0.26	0.16	0.412	0.19	− 0.21 to 0.53
Concomitant medication												
No stimulant	**0.13**	**0.040**	**0.06**	**0.00 to 0.25**	**0.38**	**0.007**	**0.14**	− **0.11 to 0.66**	**0.43**	** < 0.001**	**0.10**	**0.24 to 0.62**
No other medication	0.09	0.267	0.08	− 0.07 to 0.25	0.12	0.543	0.22	− 0.31 to 0.55	0.01	0.948	0.16	− 0.30 to 0.32
Ethnicity (Dutch)	− 0.04	0.709	0.06	− 0.16 to 0.08	0.03	0.842	0.16	− 0.29 to 0.35	0.27	0.285	0.25	− 0.24 to 0.79
Sport	− 0.05	0.310	0.05	− 0.15 to 0.05	0.01	0.949	0.15	− 0.28 to 0.30	− 0.10	0.616	0.20	− 0.48 to 0.29
IQ < 70	0.01	0.829	0.06	− 0.10 to 0.12	0.04	0.779	0.14	− 0.24 to 0.32	− 0.22	0.261	0.20	− 0.61 to 0.16
Model 2												
Dose equivalent/kg	− 0.05	0.518	0.08	− 0.21 to 0.11	0.01	0.947	0.17	− 0.31 to 0.34	− 0.08	0.079	0.05	− 0.17 to 0.01
× Duration of use (weeks)	**0.02**	**0.011**	**0.01**	**0.01 **− **0.04**	0.00	0.792	0.00	− 0.01 to 0.01	0.00	0.880	0.00	0.00 to 0.00
Baseline BMI *z *score	**1.00**	** < 0.001**	**0.02**	**0.96 **− **1.04**	**0.85**	** < 0.001**	**0.11**	**0.63 to 1.06**	**0.68**	** < 0.001**	**0.07**	**0.53 to 0.82**
× Duration of use (weeks)	− **0.01**	** < 0.001**	**0.00**	− **0.02 to 0.01**	0.00	0.667	0.00	− 0.01 to 0.01	0.00	0.695	0.00	0.00 to 0.00
Sex (male)	0.07	0.284	0.07	− 0.06 − 0.2	0.16	0.695	0.40	− 0.63 to 0.94	0.29	0.221	0.24	− 0.17 to 0.75
× Duration of use (weeks)	0.01	0.264	0.01	− 0.01 to 0.02	0.00	0.04	0.01	− 0.02 to 0.03	0.00	0.623	0.00	0.00 to 0.00
No previous antipsych. Drug	0.02	0.688	0.06	− 0.09 to 1.14	0.10	0.31	0.746	− 0.5 to 0.7	0.23	0.262	0.21	− 0.17 to 0.64
× Duration of use (weeks)	**0.02**	**0.018**	**0.01**	**0.00 to 0.03**	0.00	0.859	0.01	− 0.02 to 0.02	0.00	0.141	0.00	0.00 to 0.00
No stimulant	**0.16**	**0.033**	**0.07**	**0.01 to 0.30**	0.49	0.114	0.31	− 0.12 to 1.10	0.17	0.259	0.15	− 0.13 to 0.48
× Duration of use (weeks)	0.00	0.915	0.00	− 0.02 to 0.02	0.00	0.722	0.01	− 0.02 to 0.02	0.00	0.201	0.00	0.00 to 0.00

*Antipsych* antipsychotic, *Class *classification, *SE *standard error, *95% CI* 95% confidence interval

^a^ < 15 weeks: *n* = 144 patients, 15–52 weeks 111 patients, > 52 weeks: 58 patients

These variables were combined in the final multivariate model per time frame. The mean BMI *z *score gain per week was higher in the first 15 weeks (duration of use *β* = 0.02, 95% CI 0.01–0.03, *p* = 0.002) than between 15 and 52 weeks (*β* = 0.01, 95% CI − 0.01 to 0.02, *p* = 0.378) and after 52 weeks (*β* = 0.00, 95% CI 0.00–0.00, *p* = 0.01). During the first 15 weeks of follow-up, previous antipsychotic treatment was associated with less weight gain per week. A higher baseline BMI *z *score was found to be strongly predictive for a higher BMI *z *score during all times frames of follow-up, as was the absence of stimulant use after 15 weeks. Removal of dose equivalent and its interaction term did not remarkably change the findings of the final model. The results of the final model are shown in Table 3.

**Table 3:** Predictors of BMI *z *score in children and adolescents during antipsychotic treatment—final model

Model 3	< 15 weeks follow-up^a^				15–52 weeks follow-up^a^				> 52 weeks follow-up^a^			
	*β*	*p* value	SE	95% CI	*β*	*p* value	SE	95% CI	*β*	*p* value	SE	95% CI
Dose equivalent/kg	− 0.02	0.812	0.08	− 0.17 to 0.13	0.01	0.941	0.17	− 0.31 to 0.34	− 0.05	0.295	0.05	− 0.14 to 0.04
× Duration of use (weeks)	0.01	0.127	0.01	0.00 to 0.03	0.00	0.839	0.01	− 0.01 to 0.01	0.00	0.826	0.00	0.00 to 0.00
Baseline BMI *z *score	**0.99**	** < 0.001**	**0.02**	**0.95 to 1.03**	**0.83**	** < 0.001**	**0.11**	**0.61 to 1.04**	**0.65**	** < 0.001**	**0.08**	**0.50 to 0.80**
× Duration of use (weeks)	− **0.01**	** < 0.001**	**0.00**	− **0.02 to 0.01**	0.00	0.565	0.00	− 0.01 to 0.00	0.00	0.322	0.00	0.00 to 0.00
Previous antipsych. Drug (no)	0.00	0.975	0.06	− 0.11 to 0.11	0.09	0.782	0.31	− 0.52 to 0.69	0.22	0.284	0.20	− 0.18 to 0.62
× Duration of use (weeks)	**0.02**	**0.003**	**0.01**	**0.01 to 0.03**	0.00	0.994	0.01	− 0.02 to 0.02	0.00	0.301	0.00	0.00 to 0.00
No stimulant	0.10	0.074	0.06	− 0.01 to 0.21	**0.38**	**0.009**	**0.15**	**0.09 to 0.67**	**0.35**	** < 0.001**	**0.10**	**0.16 to 0.55**
× Duration of use (weeks)	**0.02**	**0.002**	**0.01**	**0.01 to 0.03**	0.01	0.378	0.01	− 0.01 to 0.02	**0.00**	**0.010**	**0.00**	**0.00 to 0.00**

^a^ < 15 weeks: *n* = 144 patients, 15–52 weeks 111 patients, > 52 weeks: 58 patients

## Discussion

We found that antipsychotic-induced weight gain in children and adolescents was most pronounced during the first 15 weeks of use. A higher baseline BMI *z *score predicted a higher BMI *z *score during follow-up, while the use of stimulants was associated with lower BMI *z *scores after 15 weeks. Previous antipsychotic treatment was associated with less weight gain during the first 15 weeks of treatment.

Although significant weight gain in children and adolescents on antipsychotic treatment has been widely recognised, the time course of this weight gain is only poorly documented [2]. A previous study has found that BMI *z *score gain is most pronounced during the first month of treatment, while in our cohort, a longer period of accelerated weight gain of almost 4 months was found [14]. Furthermore, although long-term data are generally lacking, several studies have described a slight decrease in BMI *z *score after 6 months [9, 14]. This plateauing was also observed in our study, but considerably later. Despite this, the BMI *z *score generally does not return to the baseline value during long-term antipsychotic use. Limited data, however, have shown that after antipsychotic discontinuation, baseline BMI *z *score values might recover [24].

The mechanisms behind this course of antipsychotic-induced weight gain in youths are complex and only partly understood. Both increased appetite, increased food intake and an altered metabolism contribute to an imbalance between energy intake and energy expenditure, regulated by both neurotransmitter systems and hormonal changes. Within the neurotransmitter systems, the interaction of antipsychotic drugs with both dopamine, histamine and serotonin receptors has been suggested to moderate weight gain [17]. These neurotransmitter–receptor interactions are expected to happen directly, as neurotransmitter-induced side effects like extrapyramidal symptoms and sedation can arise in several hours after initiation of antipsychotic treatment. As such, the weight-moderating effect too might start immediately. Conversely, the effects of dysregulation of the hormonal system are likely to take longer. Antipsychotic drugs affect leptin, also known as the satiety hormone [16, 17]. As this hormone is secreted by adipose tissue, the effects on appetite might only become apparent after an increase in adipocytes, and thus might need more time. Although both mechanisms are likely to reach a new homeostasis after a while, the different time courses of the induction of these two mechanisms might partly explain the non-linear development of weight gain in children and adolescents using antipsychotics. Nevertheless, numerous other mechanisms, including gene–environment interactions and neuropeptides, play a role and have not yet been clarified, especially in children and adolescents.

This study found that patients with higher BMI *z *score at baseline also had higher BMI *z *scores during antipsychotic treatment. However, the interaction with time suggests a faster increase in bodyweight during the first 15 weeks of treatment in children with lower bodyweight. This resembles the findings from previous, mostly short-term studies, identifying lower baseline BMI as risk factor for weight gain in children and adolescents using antipsychotics [8, 11, 12]. Nevertheless, this effect can be overestimated by the phenomenon of ‘regression to the mean’, indicating that extreme BMI values are naturally expected to grow closer to the population mean during follow-up [25]. Also, a low baseline BMI as predictor is easily confounded by stage of illness, as younger and, thus, lighter children are likely to have less prior antipsychotic exposure [16]. These children may gain more weight, as was found in our study.

Regardless of the change in weight, the finding that overweight children and adolescents are likely to remain overweight during antipsychotic treatment has important implications. Apart from the evident risk of lipid and glucose disturbances with obesity, an additional, distinct mechanism inducing metabolic abnormalities independently of weight gain is suggested for antipsychotic drugs [17]. This results in an increased prevalence of metabolic abnormalities in overweight children using antipsychotics [12], which calls for a close monitoring of overweight children at start of antipsychotic treatment. However, not all monitoring guidelines for children and adolescents on antipsychotic treatment provide such a standardized intensified monitoring schedule for overweight children [1].

Most antipsychotic-induced side effects monitoring guidelines, developed as a result of the growing awareness of cardiometabolic adverse effects of antipsychotic drugs in children and adolescents, recommend a first visit at 3 months after start [1, 26]. Given our findings that the most accelerated weight gain occurs in the first 15 weeks, it should be considered to bring this visit forward to 1 month after start of treatment. It has been shown previously that the weight gain at 1 month is predictive of problematic weight gain after 3 months in adolescents using antipsychotics, further confirming the added value of an early monitoring visit [27]. In addition, bodyweight controlling strategies are expected to be most beneficial when offered at an early stage, as childhood obesity is likely to persist in adulthood [28].

Stimulant use was associated with lower BMI *z *scores from 15 weeks of follow-up. It is well-known that decreased appetite is a common side effect of stimulants [29], thereby often leading to weight loss in children using this type of drugs. It seems obvious that concomitant stimulant use can attenuate antipsychotic-induced weight gain, although this has not been consistently demonstrated in previous studies [12, 13]. Possibly, children in our study received higher dosages of stimulant drugs, but this could not be analysed. However, although concomitant stimulant use can possibly lower weight gain in children and adolescents using antipsychotic drugs, the risk for adverse cardiovascular adverse events remains unclear. While stimulants have been associated with an increased blood pressure and heart rate, and antipsychotics with cardiac arrhythmias, little is known about the combined cardiac risks in children and adolescents [30, 31].

Children receiving a higher dose equivalent experienced more weight gain during the first weeks of use, although this finding did not remain significant in combination with other variables. A higher dosage has been described previously as a risk factor for short-term weight gain in children and adolescents using risperidone, although findings in adults have been conflicting [10, 32, 33]. Furthermore, in this study, no difference in weight-inducing potency between different types of antipsychotic drugs was found. In randomized controlled settings, however, these differences have been clearly shown, with risperidone inducing more weight gain than aripiprazole [34]. Although the relative weight-inducing properties of pipamperone have only been limitedly described, it is likely that this antipsychotic also contributes significantly to weight gain due to its strong anti-serotonergic properties. The differences between antipsychotics can diminish in an observational settings with limited sample sized like this study, as heavier children are more likely to start treatment with antipsychotics that are associated with less weight gain, such as aripiprazole. Similarly, another relatively small retrospective study performed in Dutch children and adolescents did not show a difference in weight gain between children using risperidone or aripiprazole, while based on the literature this could have been expected [9].

The results of this study should be interpreted in the light of its limitations. First, due to the merely retrospective design, no causal relationship between patient-related factors and BMI *z *score gain could be established. Also, data were collected in a real-life, clinical setting, thereby allowing changes in antipsychotic regimens upon the physician’s discretion or patient preferences. This might have influenced the results, as dosages could be lowered or the antipsychotic drug could be stopped when a child gained too much weight or had other side effects. Moreover, drug adherence was not analysed. Lastly, the sample size of this study was relatively small and only a limited proportion of patients could be followed for several years, thereby possibly missing risk factors with a low-effect size.

However, this is the first study that describes risk factors for antipsychotic-induced weight gain in children and adolescents by distinguishing between short-, middle-, and long-term uses. Also, the follow-up duration in our cohort was considerably longer than in previous studies. Using advanced mixed-modelling techniques, we could use all weight assessments instead of only studying endpoint differences, which is especially important as weight gain was non-linear. The findings of this study can guide further, targeted monitoring of children at risk for antipsychotic-induced weight gain, thereby increasing safety of antipsychotic use in the young.
